# The association between atrial fibrillation and in-hospital outcomes in chronic kidney disease patients with acute coronary syndrome: findings from the improving care for cardiovascular disease in China-acute coronary syndrome (CCC-ACS) project

**DOI:** 10.1186/s12872-021-02125-z

**Published:** 2021-07-17

**Authors:** Lijiao Yang, Nan Ye, Guoqin Wang, Weijing Bian, Fengbo Xu, Dong Zhao, Jing Liu, Yongchen Hao, Jun Liu, Na Yang, Hong Cheng, CCC-ACS

**Affiliations:** 1 Renal Division, Beijing Anzhen Hospital, Capital Medical University, No. 2 Anzhen Street, Chao yang District, Beijing, 100029 People’s Republic of China; 2 Department of Epidemiology, Beijing AnZhen Hospital, Beijing Institute of Heart, Lung and Blood Vessel Diseases, Capital Medical University, Beijing, People’s Republic of China

**Keywords:** Atrial fibrillation, Chronic kidney disease, Acute coronary syndrome

## Abstract

**Background:**

Atrial fibrillation (AF) is the most common cardiac arrhythmia in patients with chronic kidney disease (CKD) and acute coronary syndrome (ACS). This study aimed to explore the frequency and impact of AF on clinical outcomes in CKD patients with ACS.

**Methods:**

CKD inpatients with ACS between November 2014 and December 2018 were included based on the improving care for cardiovascular disease in China-ACS (CCC-ACS) project. Included patients were divided into an AF group and a non-AF group according to the discharge diagnosis. Multivariable logistic regression was used to adjust for potential confounders.

**Results:**

A total of 16,533 CKD patients with ACS were included. A total of 1418 (8.6%) patients had clinically recognized AF during hospitalization, 654 of whom had an eGFR of 45 to < 60 ml/min/1.73 m^2^, and 764 had an estimated glomerular filtration rate (eGFR) < 45 ml/min/1.73 m^2^. Compared with the non-AF group, the AF group had a higher risk of in-hospital mortality [OR 1.250; 95% CI (1.001–1.560), *P* = 0.049] and major adverse cardiovascular events (MACEs) [OR 1.361; 95% CI (1.197–1.547), *P* < 0.001]. We also found that compared with patients with eGFR 45 to < 60 ml/min/1.73 m^2^, patients with eGFR < 45 ml/min/1.73 m^2^ had a 1.512-fold increased risk of mortality and a 1.435-fold increased risk of MACEs.

**Conclusions:**

AF was a risk factor affecting the short-term prognosis of ACS patients in the CKD population. Furthermore, the lower the eGFR, the higher the risk of in-hospital mortality and MACEs in CKD patients with ACS.

*Trial registry*: Clinicaltrial.gov, NCT02306616. Registered 29 November 2014, https://clinicaltrials.gov/ct2/show/NCT02306616?term=NCT02306616&draw=2&rank=1

**Supplementary Information:**

The online version contains supplementary material available at 10.1186/s12872-021-02125-z.

## Background

Chronic kidney disease (CKD) has become a global public health problem [1, 2]. In 2012, it was reported that the prevalence of CKD in China reached 10.8% [3]. Patients with CKD have an increased risk of cardiovascular events and mortality, due to anemia, arterial calcification and vascular endothelial dysfunction [4–7]. Moreover, patients with CKD are in a two- to three-fold higher risk of atrial fibrillation (AF) than that in the general population due to left ventricular hypertrophy, poor ventricular compliance, left ventricular dilatation and activation of the renin angiotensin system [8–10]. AF is also the most common cardiac arrhythmia in patients with acute coronary syndrome (ACS), with an incidence ranging between 5 and 23% [11–14]. We can see that the incidence of AF in CKD patients is high, and the clinical prognosis is poor. The prognosis of ACS patients with CKD is also poor. AF is a common arrhythmia in ACS patients. In CKD, the occurrence of AF and the prognosis of ACS patients cause the attention of clinicians. We though that patients with AF may show a poor prognosis in CKD patients with ACS. However, there is currently no consensus on the role of AF on the prognosis of CKD patients with ACS [15–20]. Most of the studies of AF in ACS have been done in general patients including CKD and non-CKD patients. There is little information about the association of AF with clinical outcomes across the spectrum of CKD patients. Therefore, patients with CKD experiencing ACS are under-treated for AF. Based on this, the purpose of this study was to explore the association between AF and in-hospital outcomes in CKD patients with ACS.

## Methods

This article was a retrospective study based on the Improving Care for Cardiovascular Disease in China-Acute Coronary Syndrome (CCC-ACS) project, which is a national, hospital-based quality improvement project with an ongoing database, aiming to increase adherence to ACS guidelines in China and to improve patient outcomes. The project was launched in 2014 as a collaborative initiative of the American Heart Association (AHA) and the Chinese Society of Cardiology (CSC). A total of 92,509 patients from 246 hospitals were recruited, representing the diversity of ACS care in hospitals in China. Clinical data were collected via a web-based data collection platform (Oracle Clinical Remote Data Capture, Oracle Corporation). Data elements collected in this study included patient demographics, medical history, symptoms on arrival, in-hospital treatments, discharge medications, and secondary prevention strategies. The eligible patients each month were consecutively entered into the online data reporting system before the middle of the following month after patient discharge. The following four approaches were adopted to ensure the accuracy and completeness of the data: (1) face-to-face training workshops, (2) use of a standardized online reporting tool, (3) onsite quality control, and (4) monitoring of data completeness. Details of the design and methodology of the CCC-ACS project have been published previously [21].

From November 2014 to December 2018, CKD patients with ACS from the CCC-ACS were enrolled based on the principal discharge diagnosis and laboratory testing value at admission. ACS was defined in accordance with the guidelines published by the Chinese Society of Cardiology for the diagnosis and management of patients with ST-segment-elevation myocardial infarction (STEMI) and non-ST-segment elevation (NSTE) ACS [22, 23]. CKD was defined as eGFR < 60 ml/min/1.73 m^2^. CKD severity was then categorized according to the KDIGO guidelines as follows [24]: eGFR 45 to < 60 ml/min/1.73 m^2^ and eGFR < 45 ml/min/1.73 m^2^. The included patients were divided into the AF group and the non-AF group according to the discharge diagnosis. Patients in the AF group were further divided into previous and new-onset AF based on whether they had new-onset AF during this hospitalization. Institutional review board approval was granted for the aggregate data set for research and quality improvement by the Ethics Committee of Beijing Anzhen Hospital, Capital Medical University. As this was a large population-based study, approval from the Ethics Committee of Beijing Anzhen Hospital, Capital Medical University, included a waiver of informed consent.

The diagnosis of AF was based on the discharge diagnosis, and all patients’ clinical data were collected through the Web data collection platform, which had automatic data review and query function to ensure the accuracy and integrity of data. The doctors of the CCC-ACS project partner hospitals will judge whether the ACS patients had AF during their hospitalization. The CCC-ACS project partner hospitals were all certified general hospitals. Past medical history was derived from the inquiry and record of the patient’s medical history. All the laboratory testing values were the values tested the first time after admission. The first creatinine level measured during hospitalization was used to calculate the estimated glomerular filtration rate (eGFR). eGFR was calculated with the Chronic Kidney Disease Epidemiology Collaboration equation [25].

The outcomes of this study included in-hospital all-cause mortality and major adverse cardiovascular events (MACEs). MACEs were defined as a composite of reinfarction, heart failure, cardiogenic shock, cardiac arrest, stent thrombosis, and stroke (ischemic and hemorrhagic).

Continuous variables are presented as the mean and standard deviation (SD) or median and interquartile range (IQR) when distribution and variance met the appropriate conditions. Categorical variables are presented as percentages. Comparisons between groups of continuous variables were performed by t test or Mann–Whitney U test (Kruskal–Wallis). The chi-square test was used to compare the categorical variables. A multivariate logistic regression model was used to determine the association between AF and in-hospital outcomes by controlling for potential confounders. The variables entered in the multivariate model were clinically relevant variables and those significant in univariable models. For variables with missing values, the sequential regression multiple imputation method implemented by IVEware software version 0.2 (Survey Research Center, University of Michigan, Ann Arbor, MI, USA) was used to impute the missing values. All P values were 2-tailed, and *P* < 0.05 was considered statistically significant. Statistical analyses were performed using SPSS 23.0 (SPSS Inc., Chicago, IL).

## Results

This study included 16,533 CKD (eGFR < 60 ml/min/1.73 m^2^) patients with ACS. A total of 1418 (8.6%) patients had clinically recognized AF during hospitalization, and 654 (46.1%) of these patients had an eGFR of 45 to < 60 ml/min/1.73 m^2^, and 764 (53.9%) had an eGFR < 45 ml/min/1.73 m^2^. In our study, of the 1418 patients in the AF group, 592 patients (41.75%) had previous AF and 824 patients (58.25%) had new-onset AF. Patients in the AF group were more likely to be older and have previous hypertension, heart failure, CABG and stroke (all *P* < 0.001) (Table 1).

**Table 1:** Baseline characteristics of patients with or without AF

	AF (n = 1418)	Non-AF (n = 15,115)	*P* value
Male (n, %)	787 (55.5)	9551 (63.2)	< 0.001
Age (years)	76.4 ± 9.1	71.2 ± 11.3	< 0.001
SBP (mmHg)	128.7 ± 26.9	131.0 ± 27.0	0.002
DBP (mmHg)	76.1 ± 16.5	75.9 ± 15.5	0.631
Heart rate (bpm)	87.6 ± 25.5	79.8 ± 18.9	< 0.001
Previous hypertension (n, %)	1008 (71.1)	10,104 (66.8)	0.001
Previous diabetes mellitus (n, %)	412 (29.1)	4771 (31.6)	0.051
Previous HF (n, %)	205 (14.5)	800 (5.3)	< 0.001
Previous AF (n, %)	592 (41.7)	265 (1.8)	< 0.001
Previous MI (n, %)	177 (12.5)	1745 (11.5)	0.292
Previous PCI (n, %)	123 (8.7)	1438 (9.5)	0.301
Previous CABG (n, %)	23 (1.6)	113 (0.7)	< 0.001
Previous stroke (n, %)	278 (19.6)	2121 (14.0)	< 0.001
ACS type			
STEMI (n, %)	642 (45.3)	8348 (55.2)	< 0.001
eGFR (ml/min/1.73 m^2^)	41.47 ± 13.35	42.27 ± 13.85	0.035
eGFR < 45 ml/min/1.73 m^2^ (n,%)	764 (53.9)	7392 (48.9)	0.001
LVEF (%)	50.5 ± 11.8	52.4 ± 11.7	< 0.001

*AF* atrial fibrillation, *SBP* systolic blood pressure, *DBP* diastolic blood pressure, *HF* heart failure, *MI* myocardial infarction, *PCI* percutaneous coronary intervention, *CABG* coronary artery bypass grafting, *ACS* acute coronary syndrome, *STEMI* ST-segment elevation myocardial infarction, *eGFR* estimate glomerular filtration rate, *LVEF* left ventricular ejection fraction

**Fig. 1:**
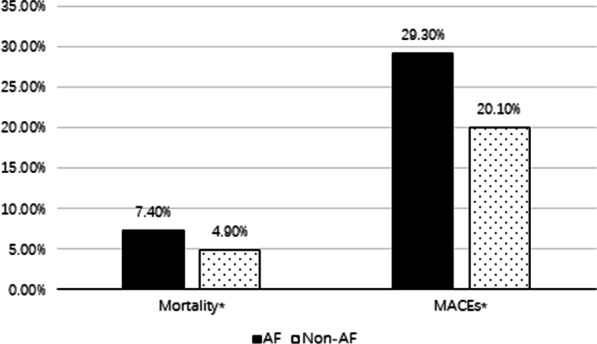
Prevalence of in-hospital outcomes in patients with or without AF. *P < 0.001. *AF* atrial fibrillation, *MACEs* major adverse cardiovascular events

In our study, the proportion of patients who received antiplatelet, warfarin, statins, beta-blockers and ACEI/ARB drugs before admission was low regardless of whether they had AF. Compared with patients without AF, patients with AF were more likely to be treated with warfarin (3.2% vs 0.2%, *P* < 0.001) and beta-blockers (12.8% vs 10.9%, *P* = 0.030), while they were less likely to receive percutaneous coronary intervention (40.9% vs 55.9%, *P* < 0.001) during hospitalization (Additional file 1: Table S1). We could also know that 71 patients (5.0%) with AF received warfarin anticoagulation during hospitalization, and 949 patients (66.9%) received subcutaneous anticoagulation therapy. As shown in Fig. 1, compared with patients without AF, patients with AF had a poorer prognosis. The proportion of mortality (7.40% vs 4.90%, *P* < 0.001) and MACEs (29.30% vs 20.10%, *P* < 0.001) were significantly higher in the AF group (Fig. 1). After adjusting for confounders, AF was significantly related to in-hospital mortality [OR 1.250; 95% CI (1.001–1.560), *P* = 0.049] (Table 2) and MACEs [OR 1.361; 95% CI (1.197–1.547), *P* < 0.001] (Table 3). Further analysis showed that new-onset AF was significantly related to in-hospital mortality and MACEs [OR 1.334; 95% CI (1.013–1.755), *P* = 0.040; OR 1.346; 95% CI (1.143–1.586), *P* < 0.001, respectively]. However, prior AF was significantly related to in-hospital MACEs [OR 1.369; 95% CI (1.129–1.661), *P* = 0.001, not related to mortality [OR 1.149; 95% CI (0.812–1.625), *P* = 0.433]. We also found that compared with the patients with eGFR 45 to < 60 ml/min/1.73 m^2^, patients with eGFR < 45 ml/min/1.73 m^2^ had a 1.512-fold increased risk of mortality (Table 2) and a 1.435-fold increased risk of MACEs (Table 3).

**Table 2:** Association between AF and in-hospital mortality in CKD patients with ACS

Risk factors	Unadjusted		Adjusted^a^	
	OR (95% CI)	*P* value	OR (95% CI)	*P* value
AF	1.545 (1.250–1.909)	< 0.001	1.250 (1.001–1.560)	0.049
eGFR(ml/min/1.73 m^2^)				
45 to < 60	1 (reference)		1 (reference)	
< 45	1.841 (1.596–2.125)	< 0.001	1.512 (1.303–1.753)	< 0.001
Age (10-year increase)	1.367 (1.280–1.461)	< 0.001	1.287 (1.201–1.379)	< 0.001
Previous diabetes mellitus	1.227 (1.066–1.413)	0.004	1.331 (1.148–1.542)	< 0.001
STEMI	1.919 (1.654–2.227)	< 0.001	2.540 (2.162–2.985)	< 0.001

*AF* atrial fibrillation, *STEMI* ST-segment elevation myocardial infarction, *eGFR* estimated glomerular filtration rate, *PCI* percutaneous coronary intervention, *ACEI* angiotensin-converting enzyme inhibitor, *ARB* angiotensin receptor blockade

^a^Adjusted for AF, age (10-year increase), sex, previous diabetes mellitus, previous heart failure, stroke, STEMI, eGFR strata, PCI, ACEI/ARB and beta-blockers use during hospitalization

**Table 3:** Association between AF and in-hospital MACEs in CKD patients with ACS

Risk factors	Unadjusted		Adjusted^a^	
	OR (95% CI)	*P* value	OR (95% CI)	*P* value
AF	1.648 (1.460–1.860)	< 0.001	1.361 (1.197–1.547)	< 0.001
eGFR (ml/min/1.73 m^2^)				
45 to < 60	1 (reference)		1 (reference)	
< 45	1.632 (1.513–1.761)	< 0.001	1.435 (1.326–1.554)	< 0.001
Age (10-year increase)	1.241 (1.199–1.284)	< 0.001	1.166 (1.125–1.210)	< 0.001
Previous diabetes mellitus	1.297 (1.201–1.401)	< 0.001	1.297 (1.195–1.406)	< 0.001
STEMI	1.372 (1.271–1.481)	< 0.001	1.897 (1.741–2.067)	< 0.001

*AF* atrial fibrillation, *STEMI* ST-segment elevation myocardial infarction, *eGFR* estimated glomerular filtration rate, *PCI* percutaneous coronary intervention, *ACEI* angiotensin-converting enzyme inhibitor, *ARB* angiotensin receptor blockade

^a^Adjusted for AF, age (10-year increase), sex, previous diabetes mellitus, previous heart failure, previous myocardial infarction, stroke, STEMI, eGFR strata, PCI, ACEI/ARB and beta-blockers use during hospitalization

## Discussion

This study was the first known study on the incidence and prognosis of ACS with concomitant AF in patients with CKD from a Chinese database. The results showed that the prevalence of ACS with concomitant AF was 8.6% in patients with CKD, which was similar to the findings of previous studies conducted in the general population (5.0–23.0%) [9–11]. In this study, AF included both new-onset AF during hospitalization and pre-existing AF because the diagnosis of AF was based on the discharge diagnosis in the database. We found that 41.7% of the cases of AF in this study were pre-existing. Our study showed that the patients with AF were more likely to be older; have comorbidities, such as previous hypertension and heart failure; and have lower baseline eGFR, which was consistent with previous studies [26–28]. In addition, the proportion of ACS patients with AF receiving PCI during hospitalization was significantly lower than that of those without AF, which was also consistent with our clinical practice. In the CKD population, the proportion of ACS patients with other cardiovascular diseases receiving PCI was lower than that of patients with ACS alone due to the greater risk of adverse events. In our study, we focused on the prognosis of patients with ACS during hospitalization, a period that has the highest risk of early clinical events [29]. Some previous studies suggested that AF can increase the risk of short-term and long-term mortality, stroke and bleeding events in ACS patients [30–32]. However, there were also studies that reported different results. Kinjo et al. analyzed the data of 2475 patients with AMI who underwent PCI within 24 h after onset. The results revealed that AF was an independent predictor of 1-year mortality but was not a predictor of in-hospital mortality [26]. Luca et al. analyzed the data of 16,803 ACS patients in Italy. The results showed that AF at admission was not an independent predictor of in-hospital mortality, although patients with AF had a higher risk of mortality than those without AF. However, the characteristics of AF and the stratified analysis of renal function were not mentioned in this study [33]. The incidence of AF in CKD patients is high, and the clinical prognosis is poor. The prognosis of ACS patients with CKD is also poor. AF is a common arrhythmia in ACS patients. In CKD, the occurrence of AF and the prognosis of ACS patients cause the attention of clinicians and should be studied.

In our study, AF was defined according to the discharge diagnosis, which made it more accurate in terms of the patients’ characteristics. The results showed that the risk of in-hospital mortality and MACEs was significantly higher in the AF group than in the non-AF group. Moreover, after adjusting for confounders, AF was found to be an independent risk factor for in-hospital mortality and MACEs in CKD patients with ACS. Further analysis showed that new-onset AF was significantly related to in-hospital mortality and MACEs. However, prior AF was significantly related to in-hospital MACEs, not related to mortality. Furthermore, we analyzed the relationship between renal function and in-hospital prognosis in CKD patients with ACS. The results showed that the decrease in renal function was an independent risk factor for adverse in-hospital outcomes in CKD patients with ACS. These patients, especially those with severe renal insufficiency, should be given more attention and closer monitoring in clinical practice to reduce the incidence of in-hospital mortality and MACEs.

Our study also had some limitations. First, since this study included observational data from a large database, although we adjusted for a wide range of potential confounders, we cannot rule out residual confounding factors, including whether the AF was new-onset or pre-existing and the type, occurrence time and duration of AF. Second, because of the atypical symptoms of some patients with AF, there may be some cases of undiagnosed AF. Third, due to the limitation of data collection, the definition of CKD in our study only included eGFR < 60 ml/min/1.73 m^2^, which is different from the KDIGO criteria. Finally, due to the observational nature of the study, causality and the underlying mechanisms could not be determined.

## Conclusions

In summary, our study was the first and largest to investigate ACS with concomitant AF in CKD patients in China. In this study, we found that AF was a risk factor affecting the short-term prognosis of ACS patients in the CKD population. Furthermore, the lower the eGFR, the higher the risk of in-hospital mortality and MACEs in CKD patients with ACS. These patients, especially those with severe renal insufficiency, should be given more attention and closer monitoring in clinical practice. Further studies are needed to investigate the effect of AF on the long-term prognosis of CKD patients with ACS.

## Supplementary Information

**Additional file 1**. Table S1. Treatments pre-hospital and during hospitalization. Table S2. Investigators of CCC-ACS project.

## Data Availability

The datasets used and analyzed during the current study are available from the principal investigator of CCC-ACS on reasonable request.

## Abbreviations

AF: Atrial fibrillation
CKD: Chronic kidney disease
ACS: Acute coronary syndrome
CCC-ACS: Care for cardiovascular disease in China-acute coronary syndrome
eGFR: Estimated glomerular filtration rate
MACEs: Major adverse cardiovascular events
AHA: American Heart Association
CSC: Chinese Society of Cardiology
STEMI: ST-segment-elevation myocardial infarction
NSTE: Non-ST-segment elevation
SD: Standard deviation
IQR: Interquartile range
HF: Heart failure
CABG: Coronary artery bypass grafting
ACEI: Angiotensin-converting enzyme inhibitor
ARB: Angiotensin receptor blockade
PCI: Percutaneous coronary intervention
SBP: Systolic blood pressure
DBP: Diastolic blood pressure
LVEF: Left ventricular ejection fraction
